# Supplementary figures and images for: Edge-focused wire arc additive manufacturing: Method development with ANN-based stress–strain and mass-efficiency
Source: PLoS One. 2026 Jun 5;21(6):e0349828. doi: 10.1371/journal.pone.0349828 (PMC13240928; doi:10.1371/journal.pone.0349828)

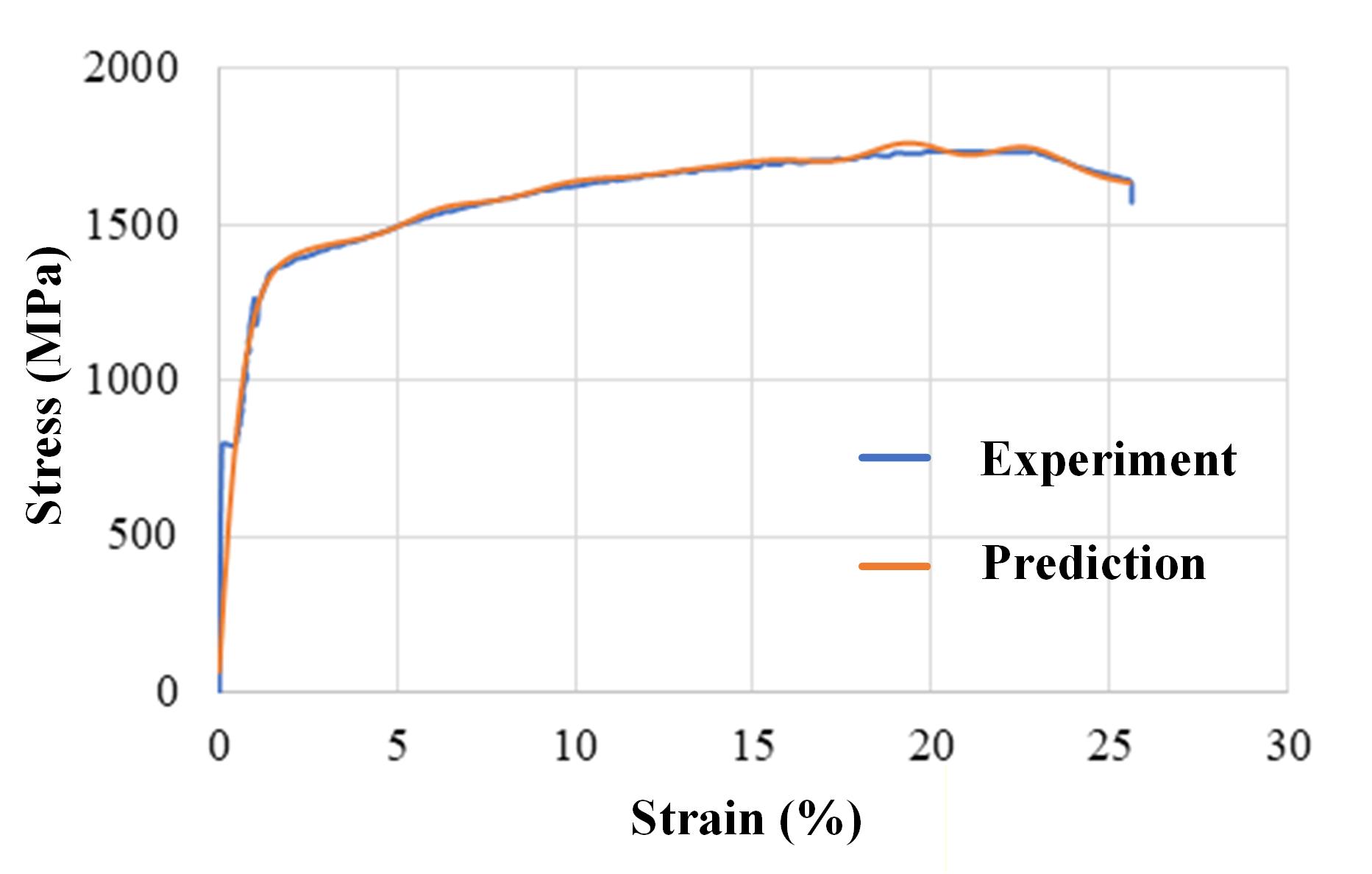

Supplement: S1 Fig — (JPG) [file pone.0349828.s001.jpg]

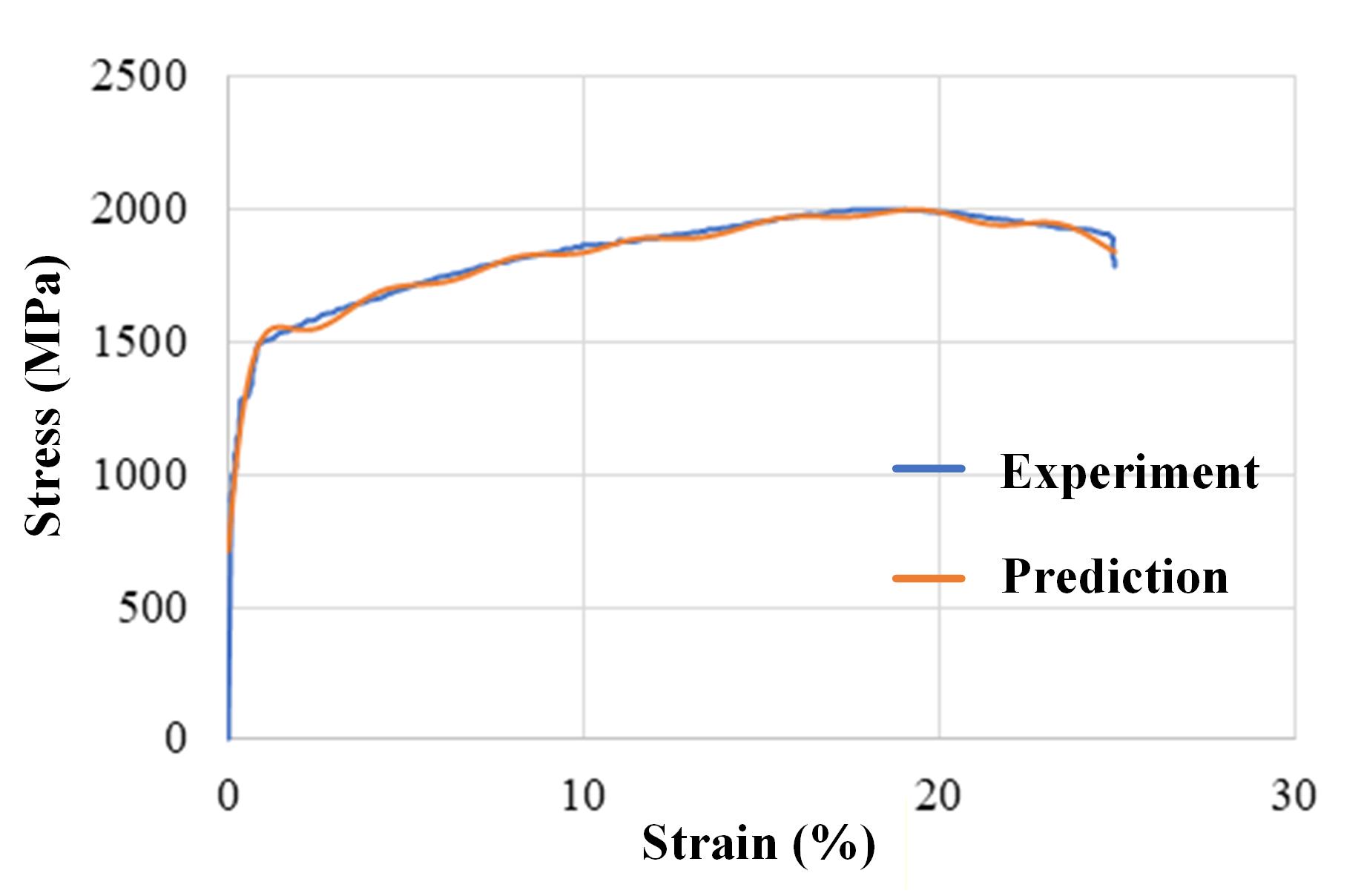

Supplement: S2 Fig — (JPG) [file pone.0349828.s002.jpg]

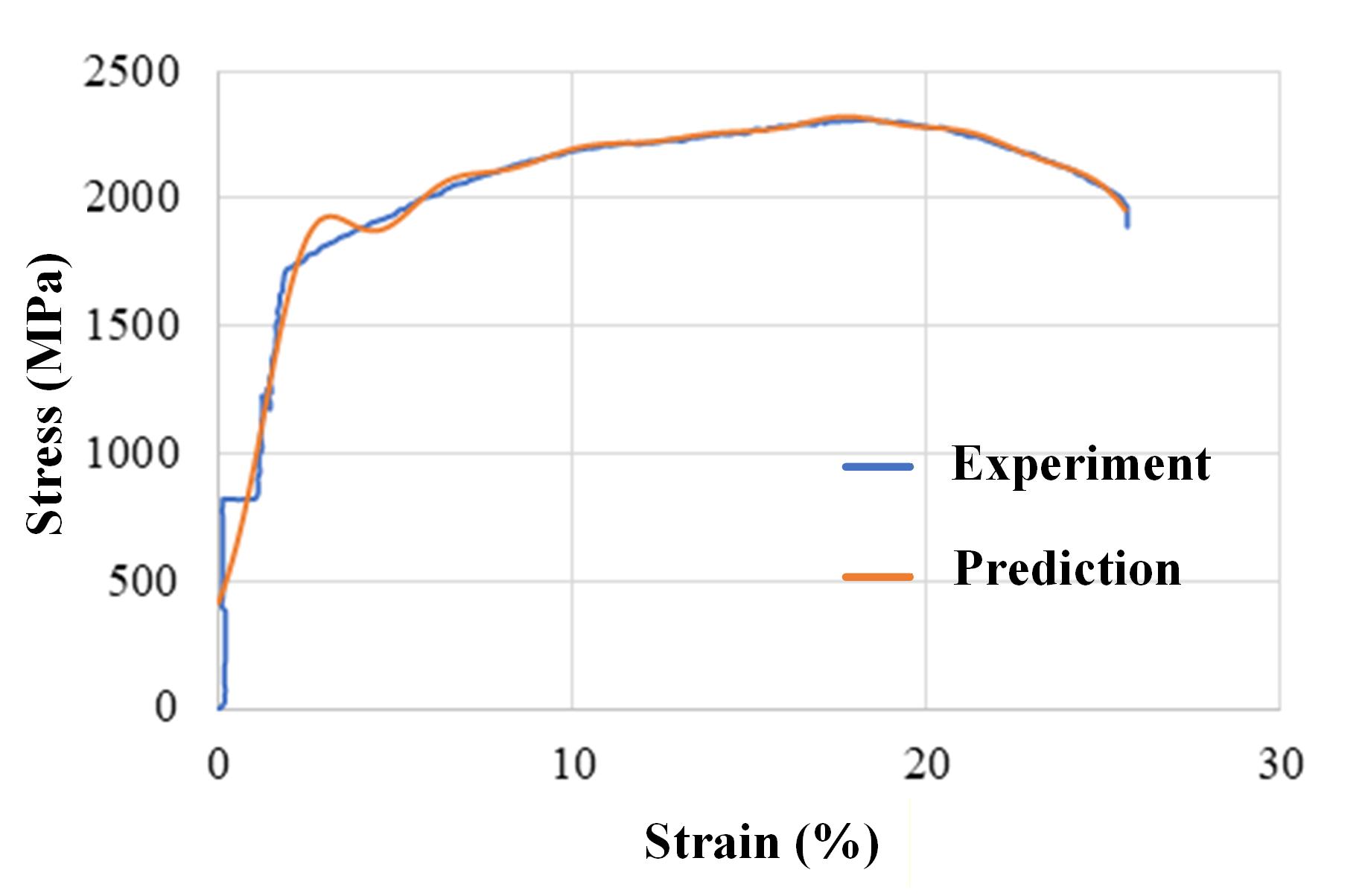

Supplement: S3 Fig — (JPG) [file pone.0349828.s003.jpg]

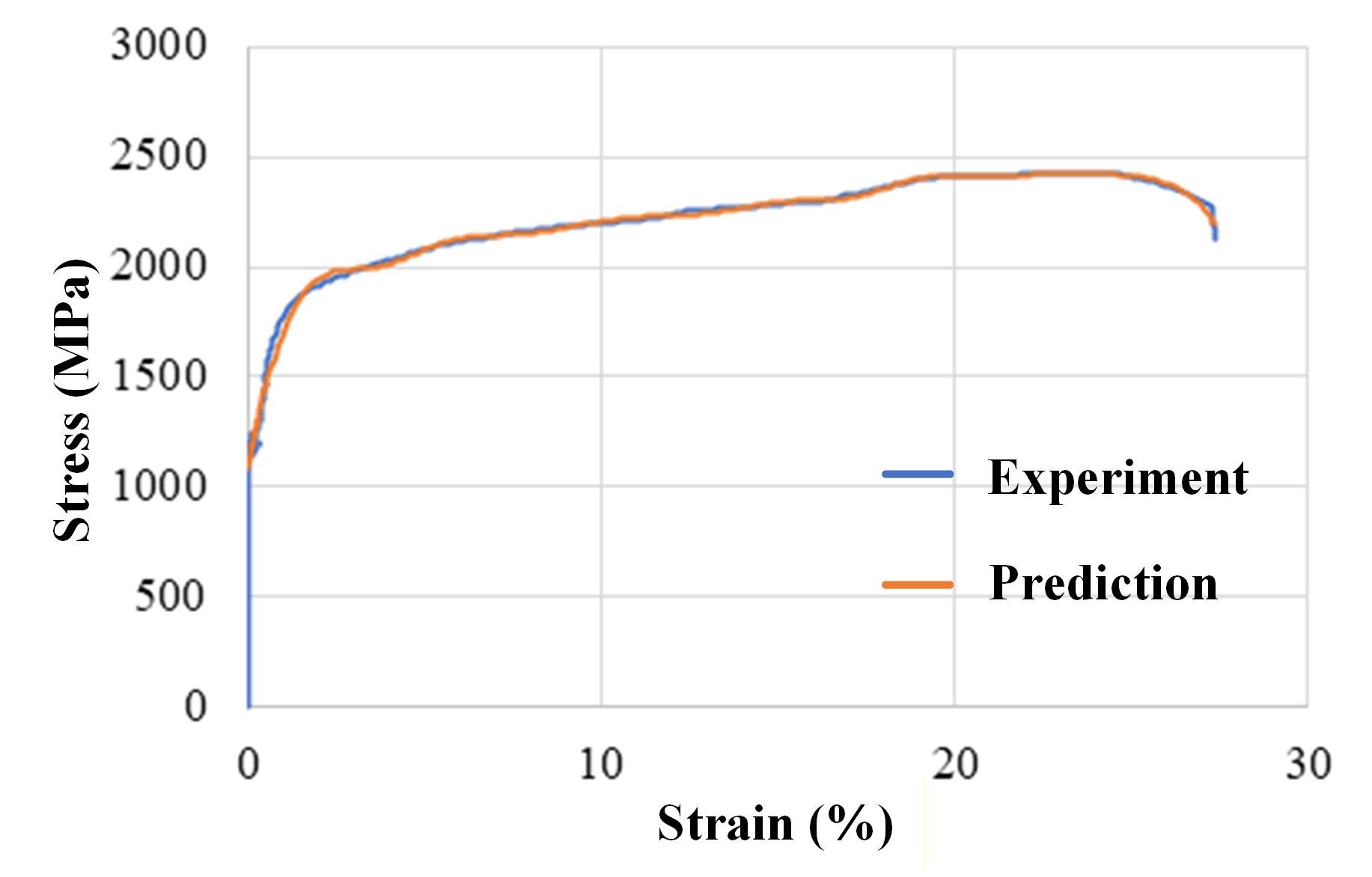

Supplement: S4 Fig — (JPG) [file pone.0349828.s004.jpg]

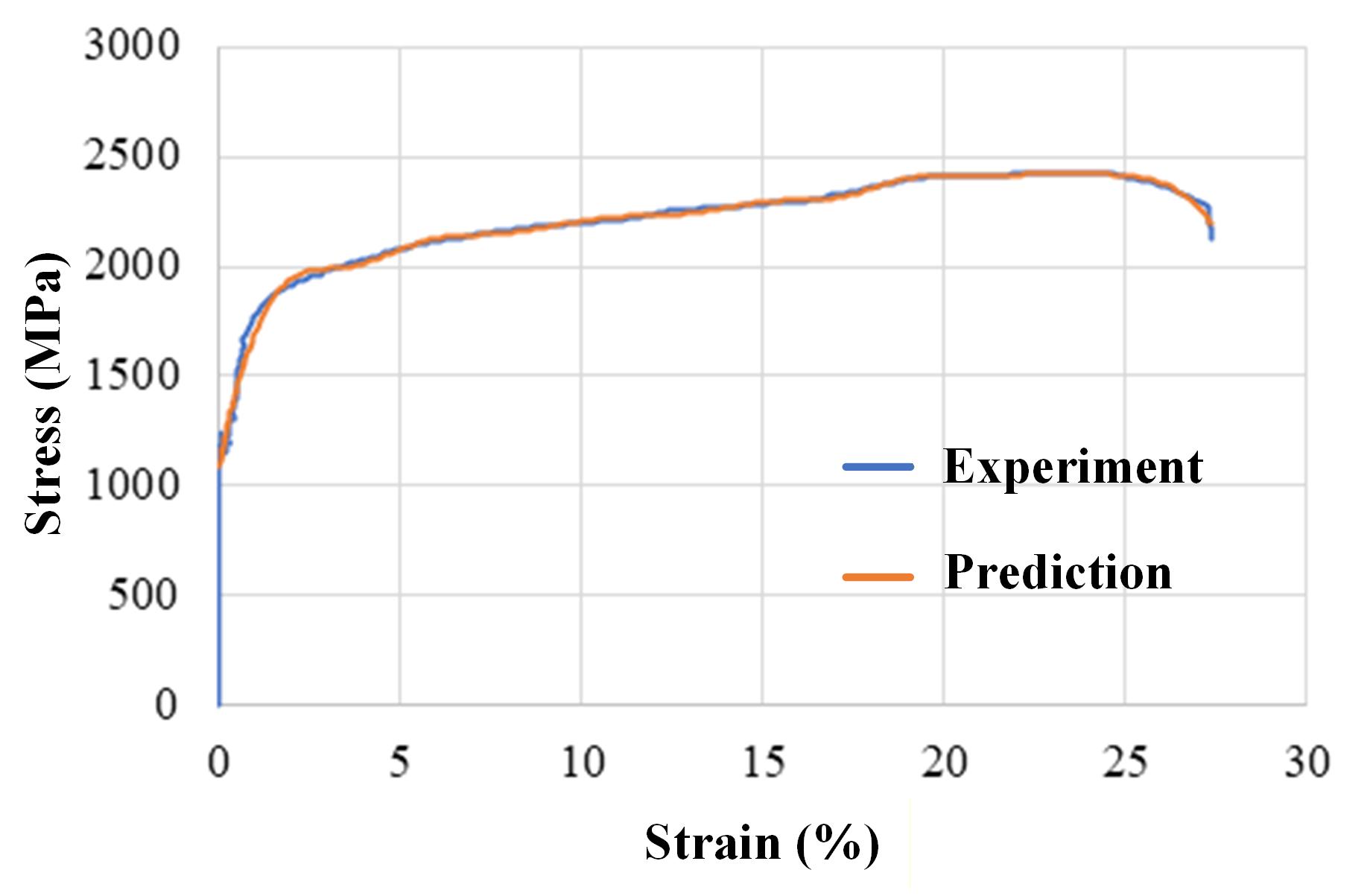

Supplement: S5 Fig — (JPG) [file pone.0349828.s005.jpg]

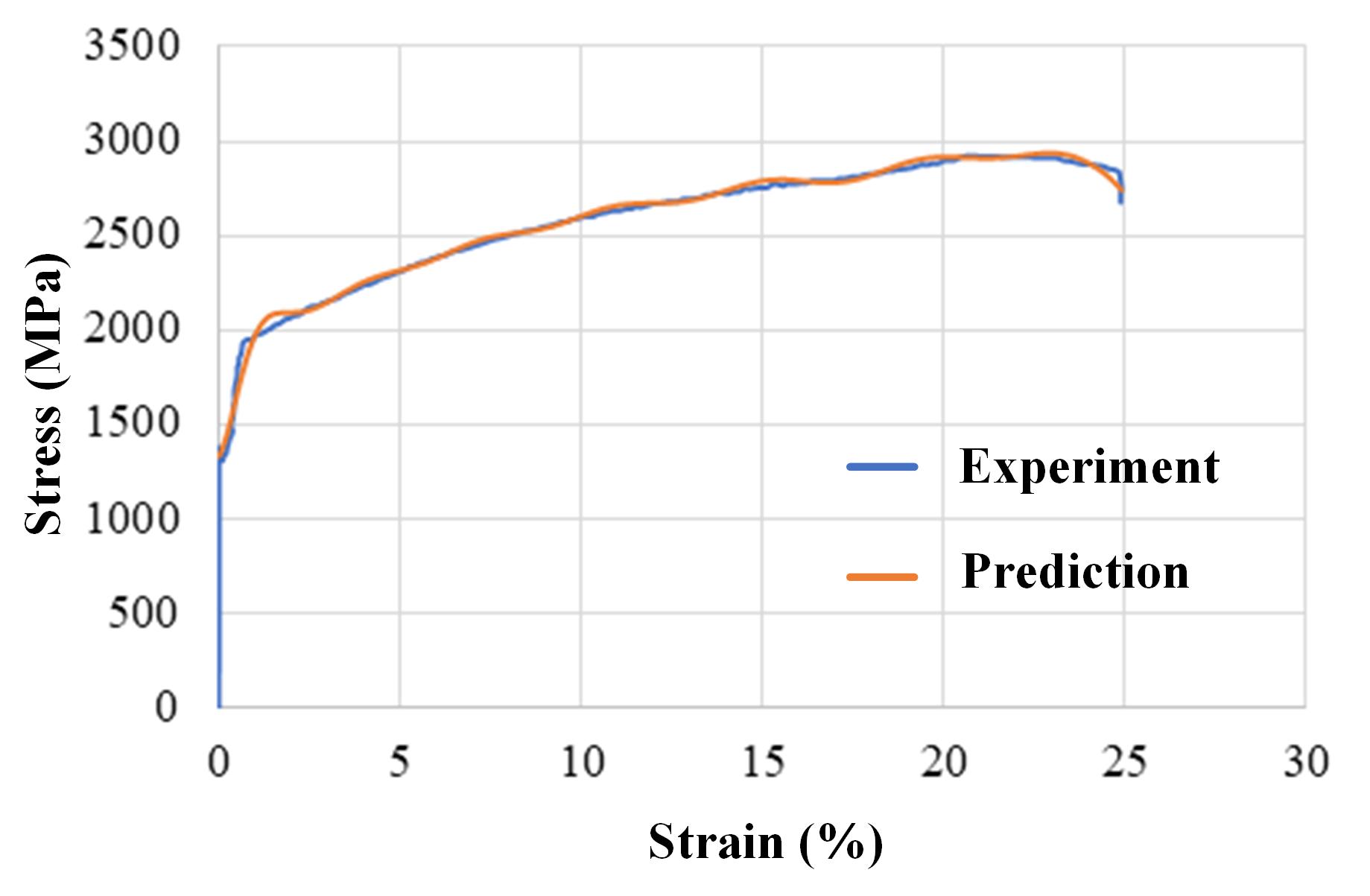

Supplement: S6 Fig — (JPG) [file pone.0349828.s006.jpg]

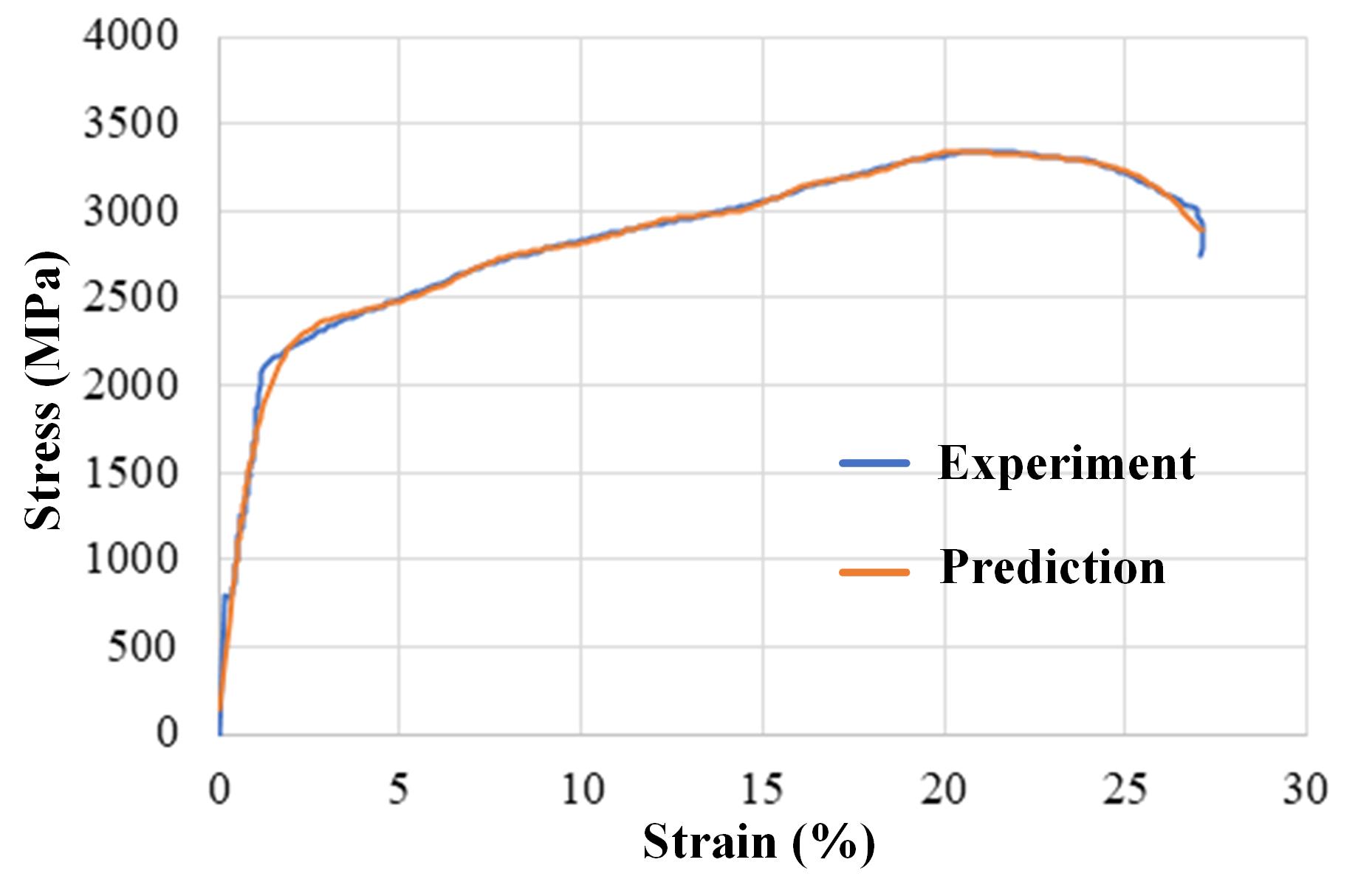

Supplement: S7 Fig — (JPG) [file pone.0349828.s007.jpg]

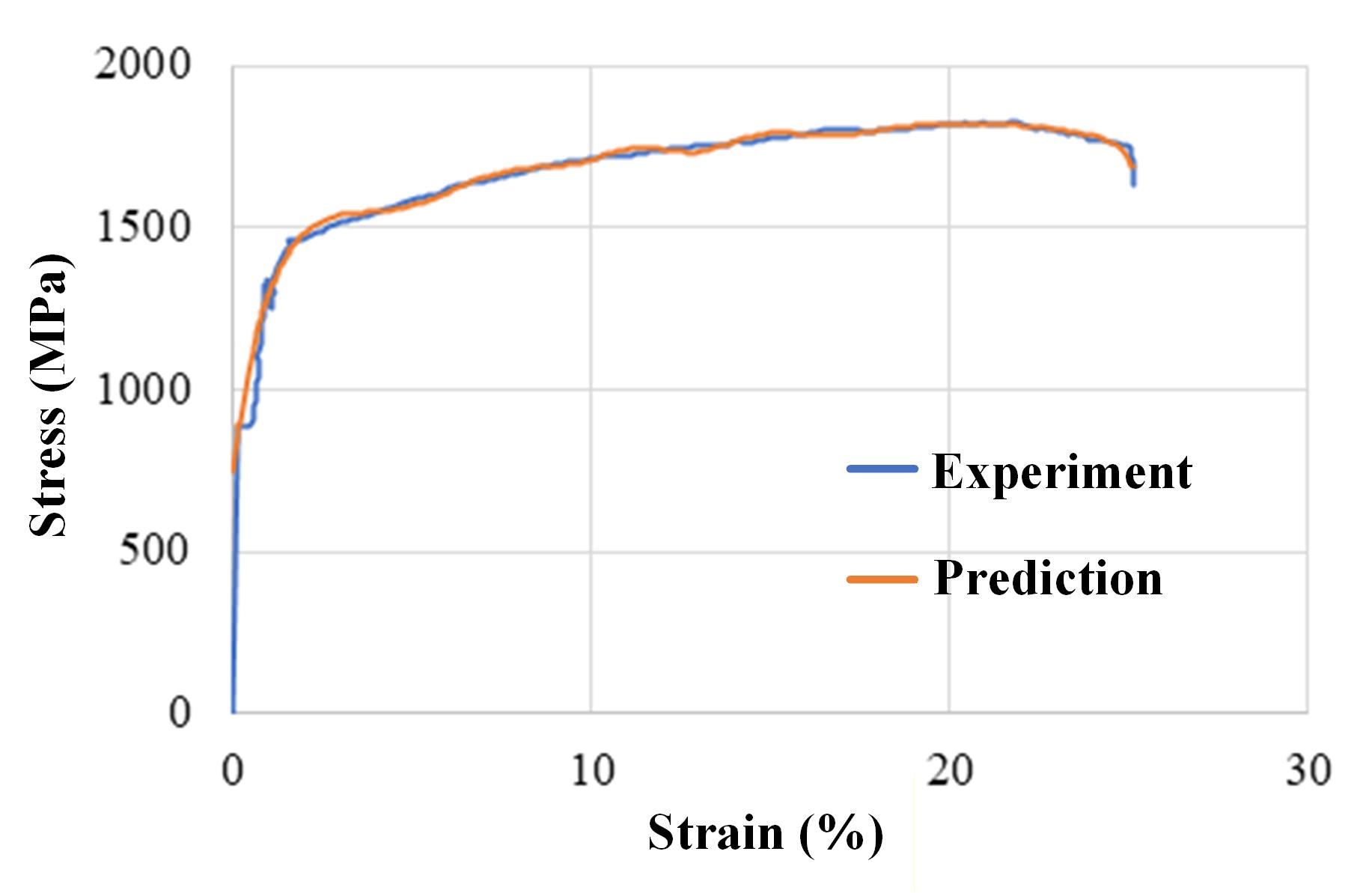

Supplement: S8 Fig — (JPG) [file pone.0349828.s008.jpg]

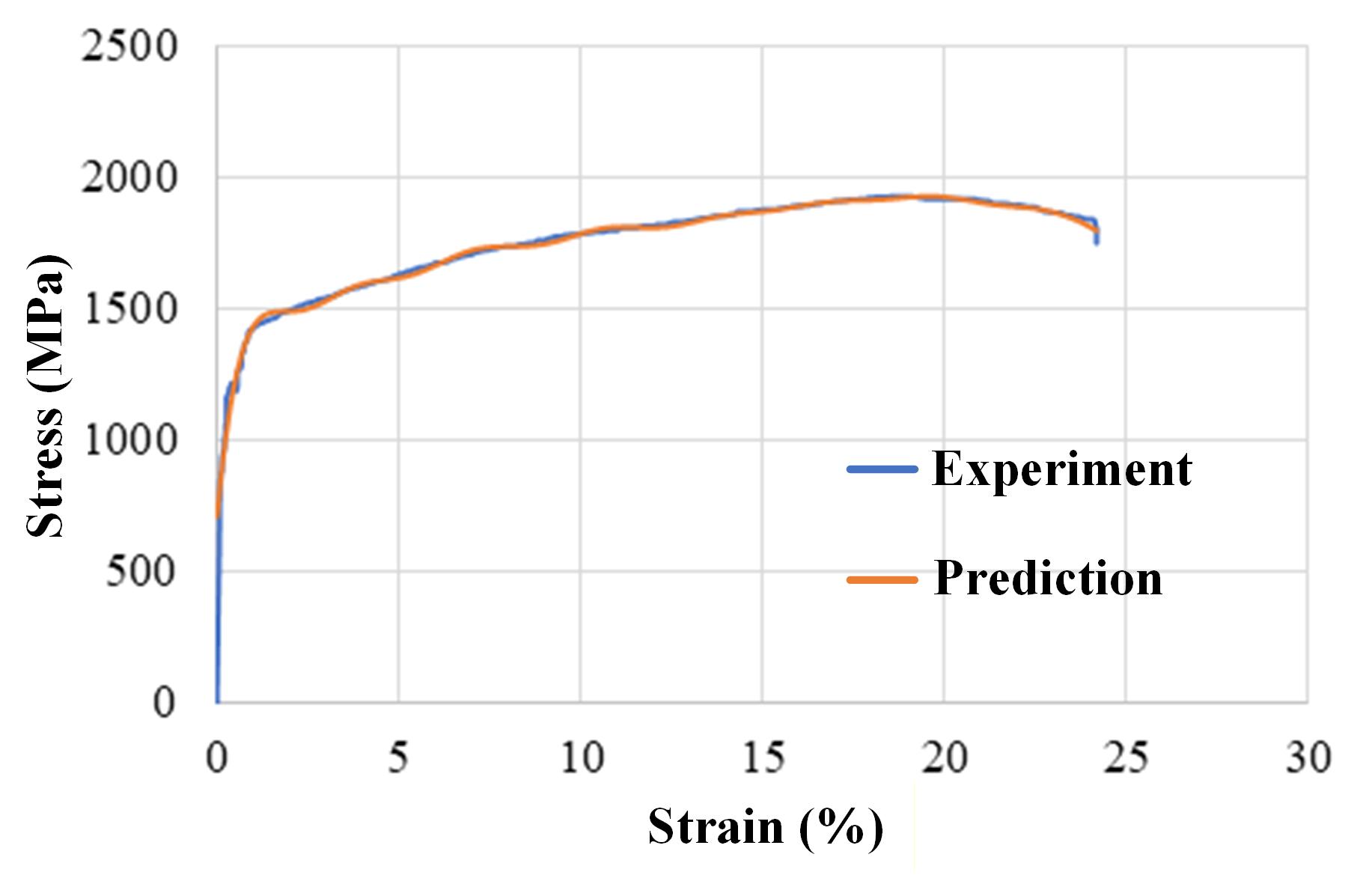

Supplement: S9 Fig — (JPG) [file pone.0349828.s009.jpg]

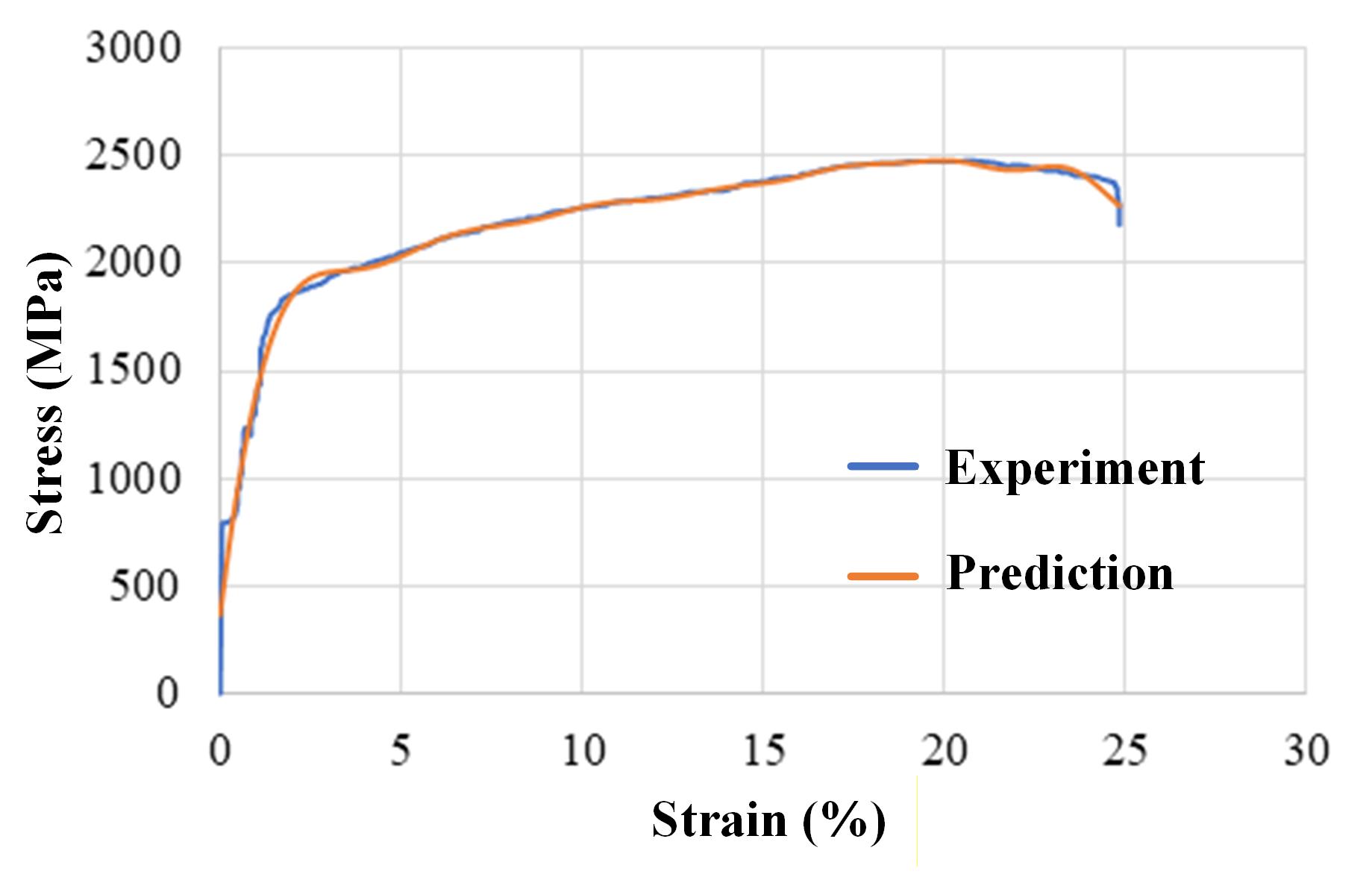

Supplement: S10 Fig — (JPG) [file pone.0349828.s010.jpg]

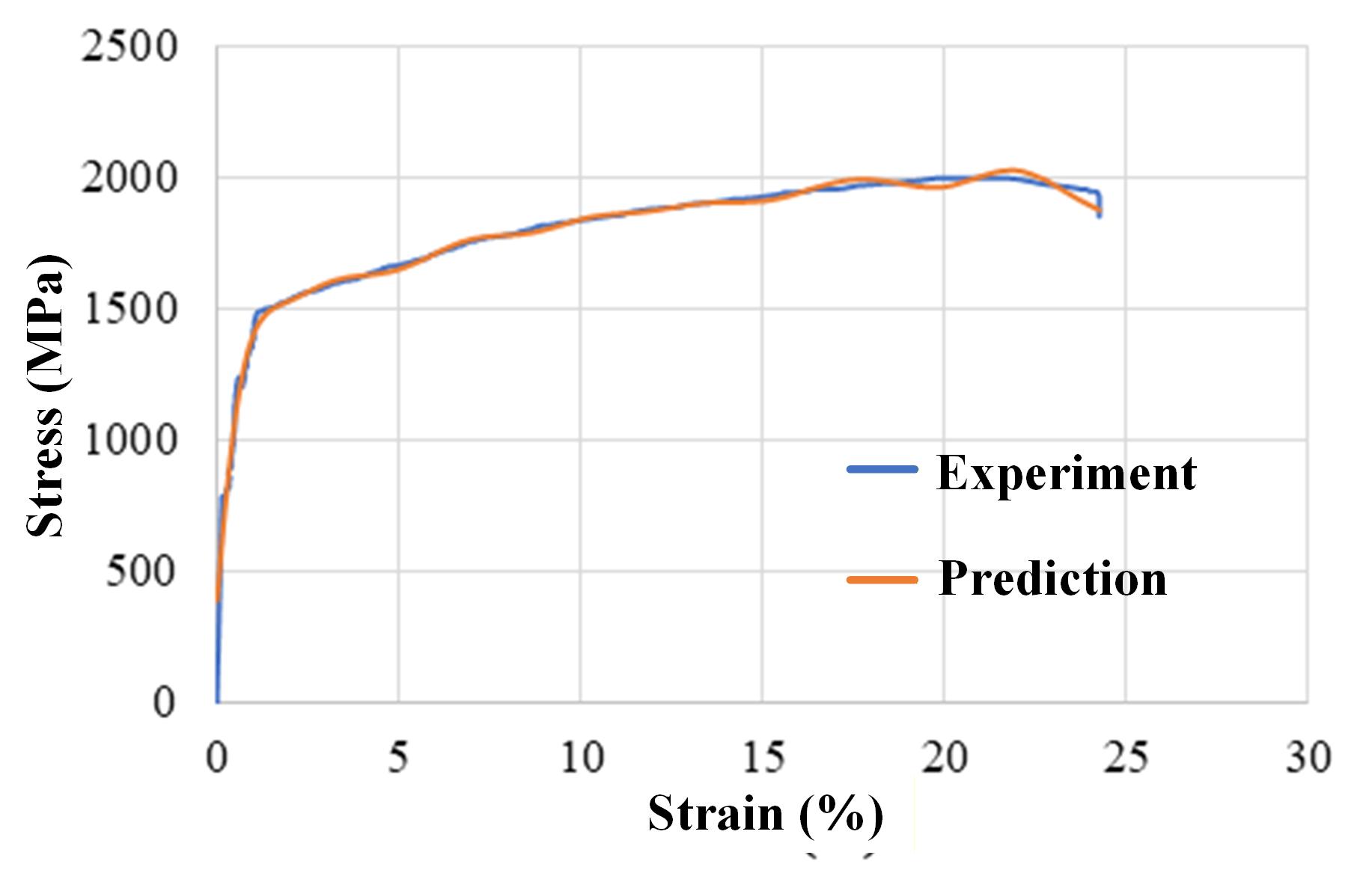

Supplement: S11 Fig — (JPG) [file pone.0349828.s011.jpg]

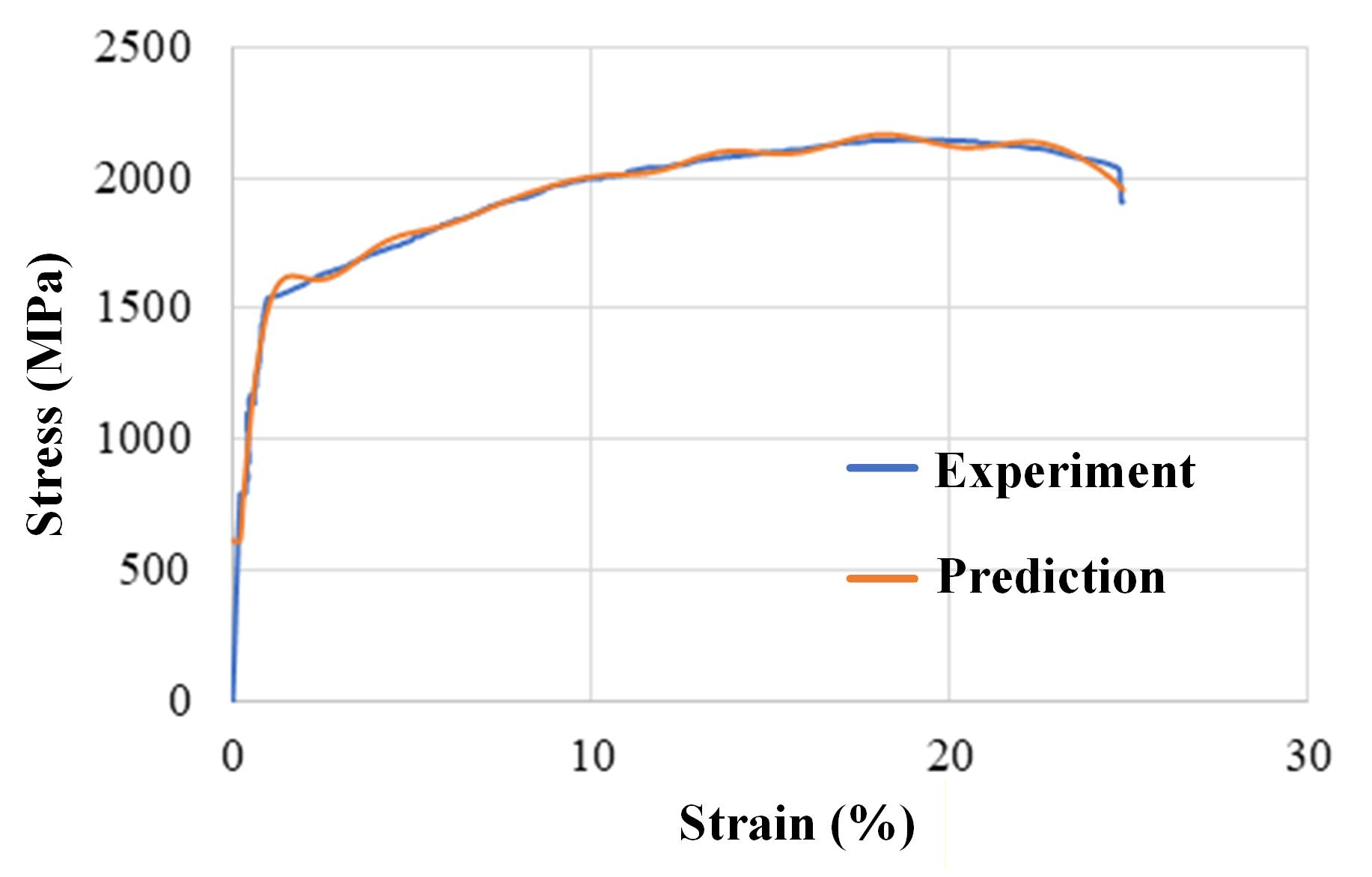

Supplement: S12 Fig — (JPG) [file pone.0349828.s012.jpg]

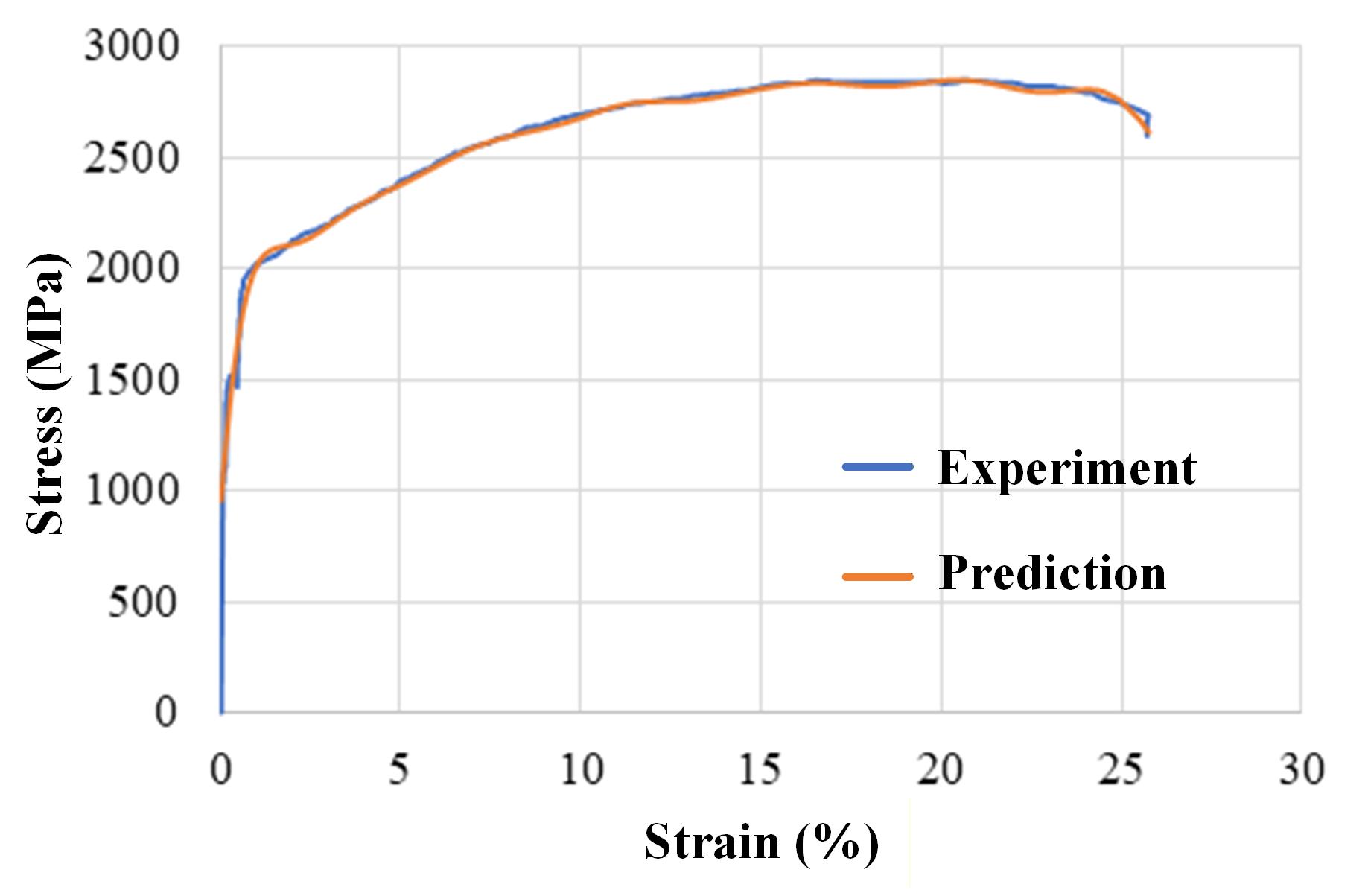

Supplement: S13 Fig — (JPG) [file pone.0349828.s013.jpg]

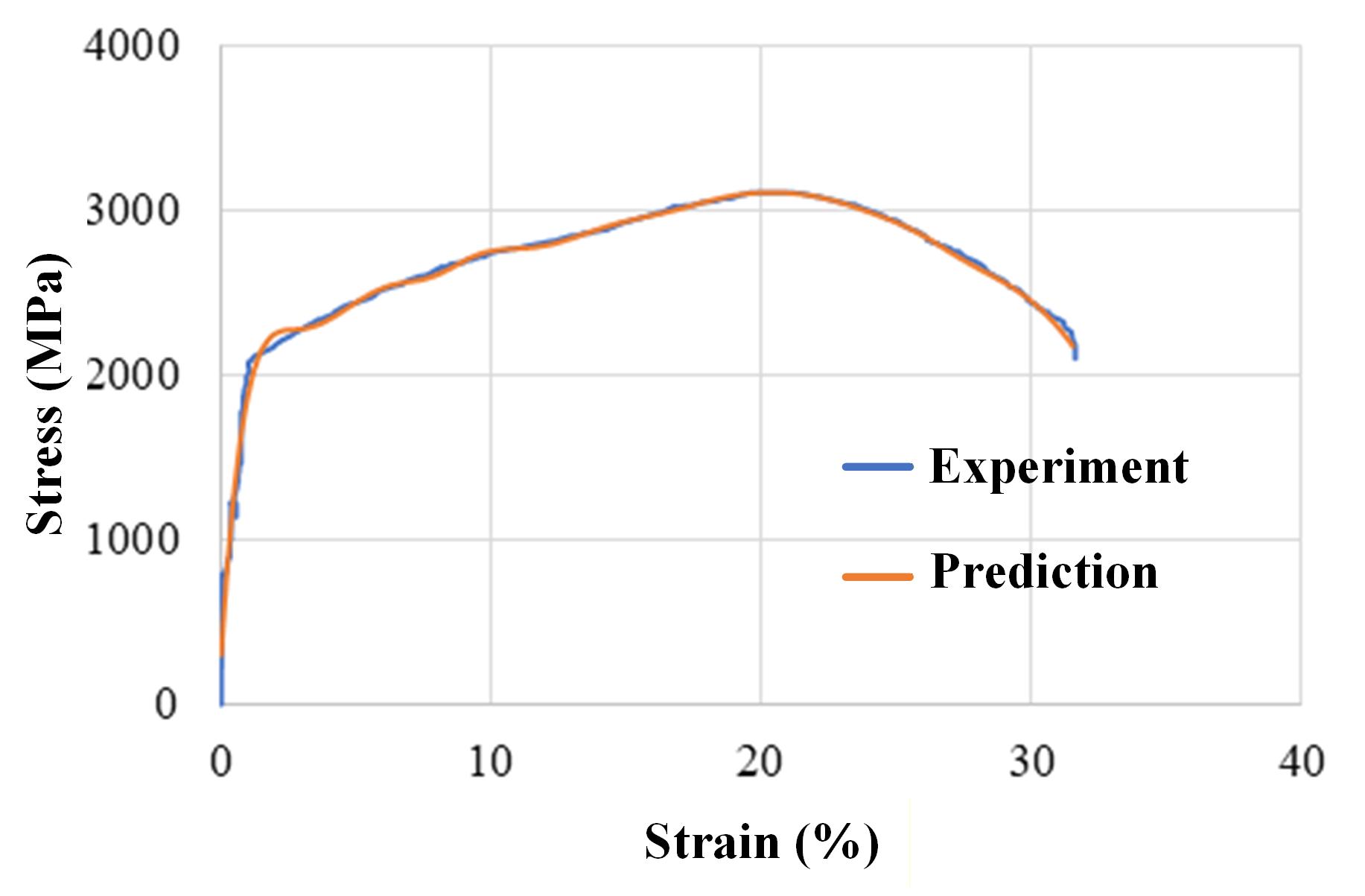

Supplement: S14 Fig — (JPG) [file pone.0349828.s014.jpg]

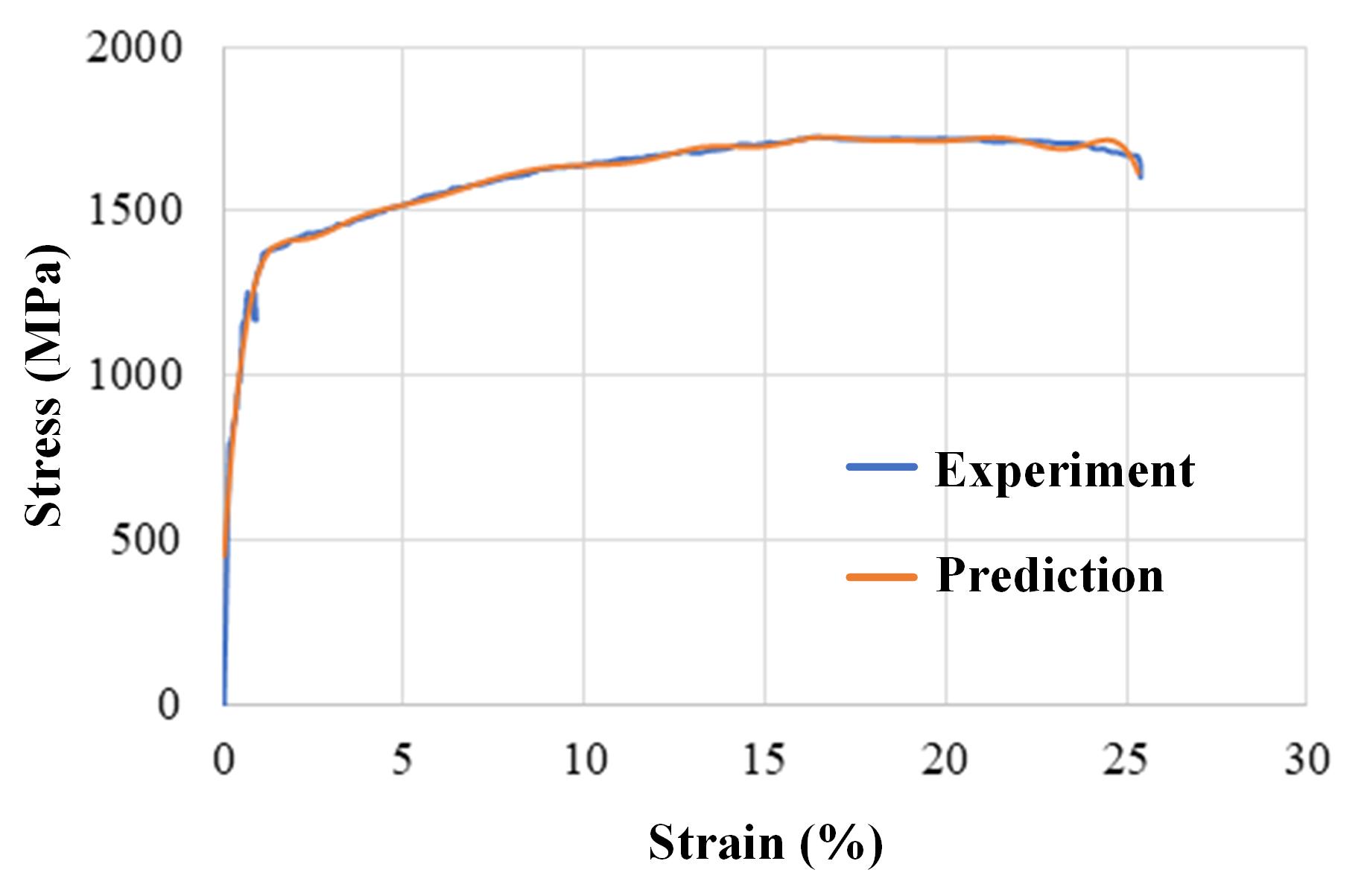

Supplement: S15 Fig — (JPG) [file pone.0349828.s015.jpg]

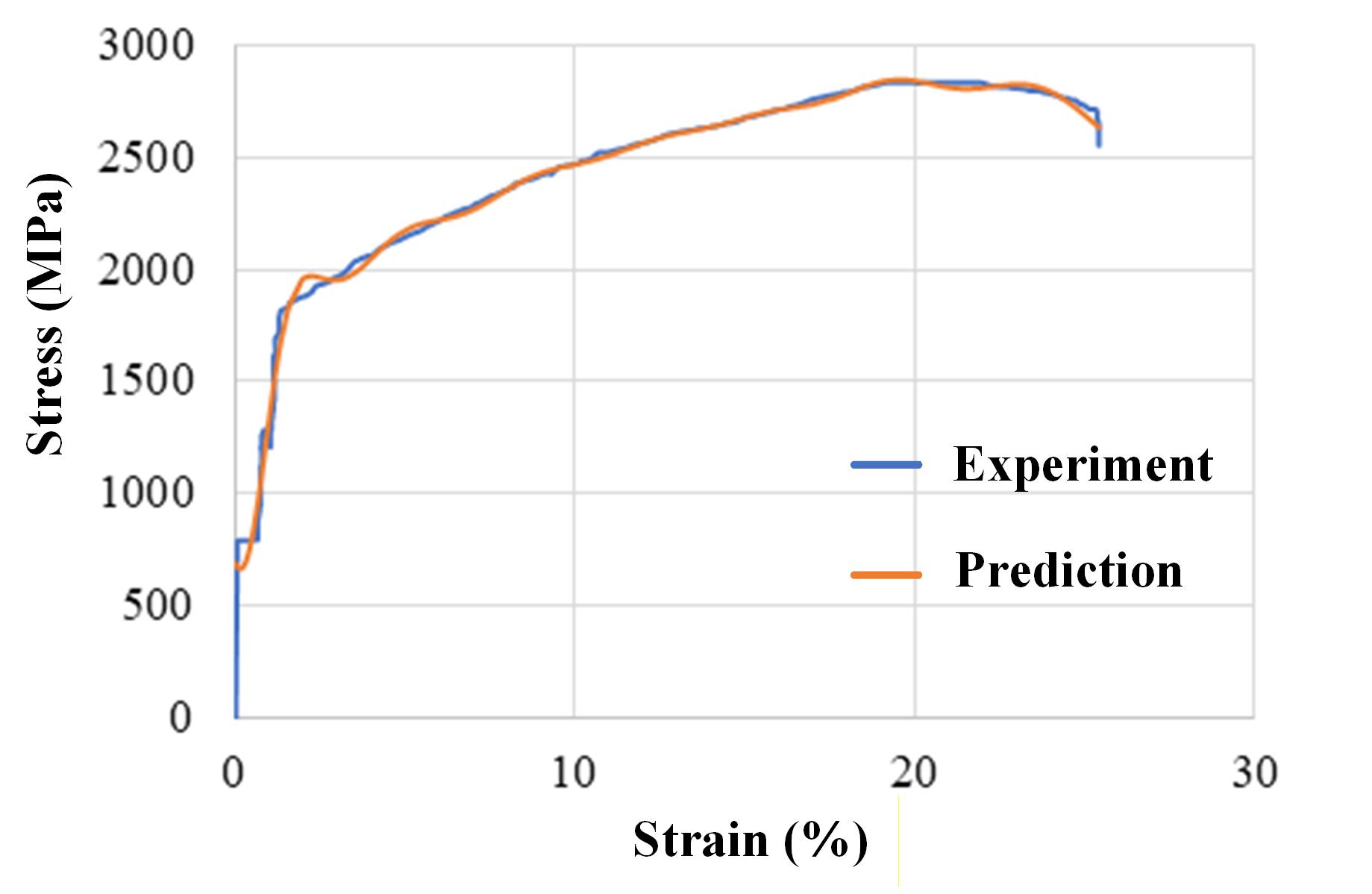

Supplement: S16 Fig — (JPG) [file pone.0349828.s016.jpg]

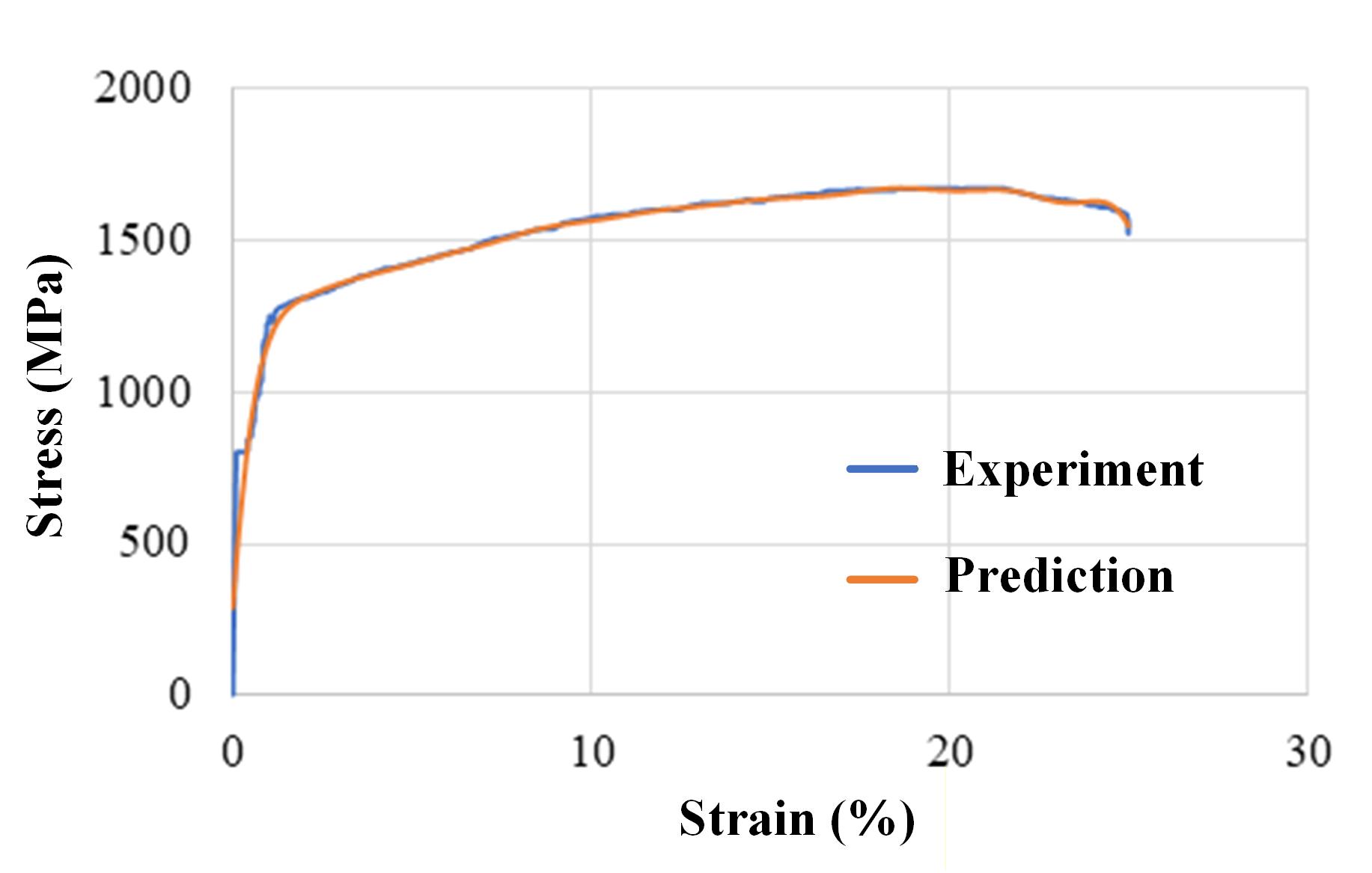

Supplement: S17 Fig — (JPG) [file pone.0349828.s017.jpg]

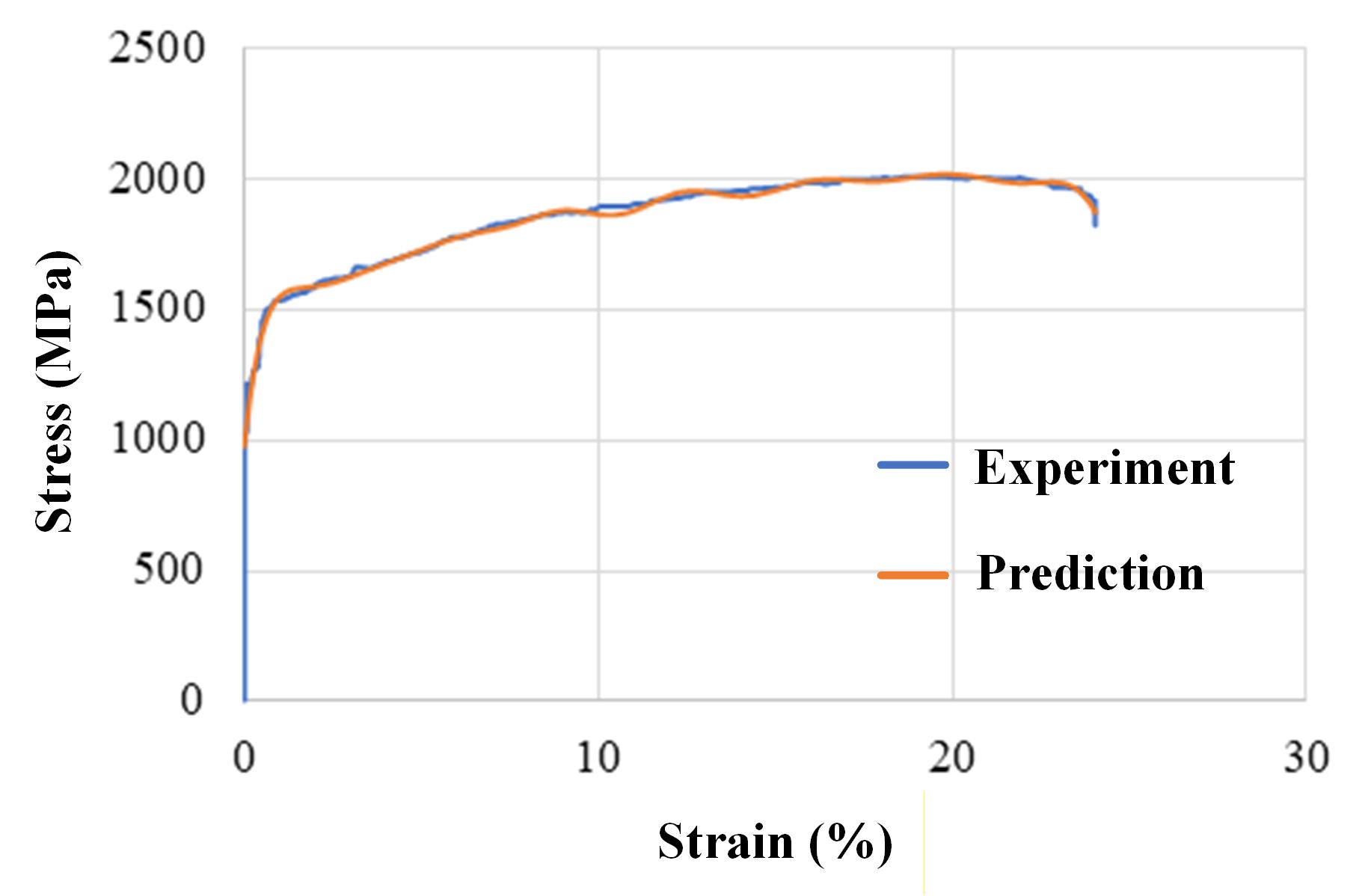

Supplement: S18 Fig — (JPG) [file pone.0349828.s018.jpg]

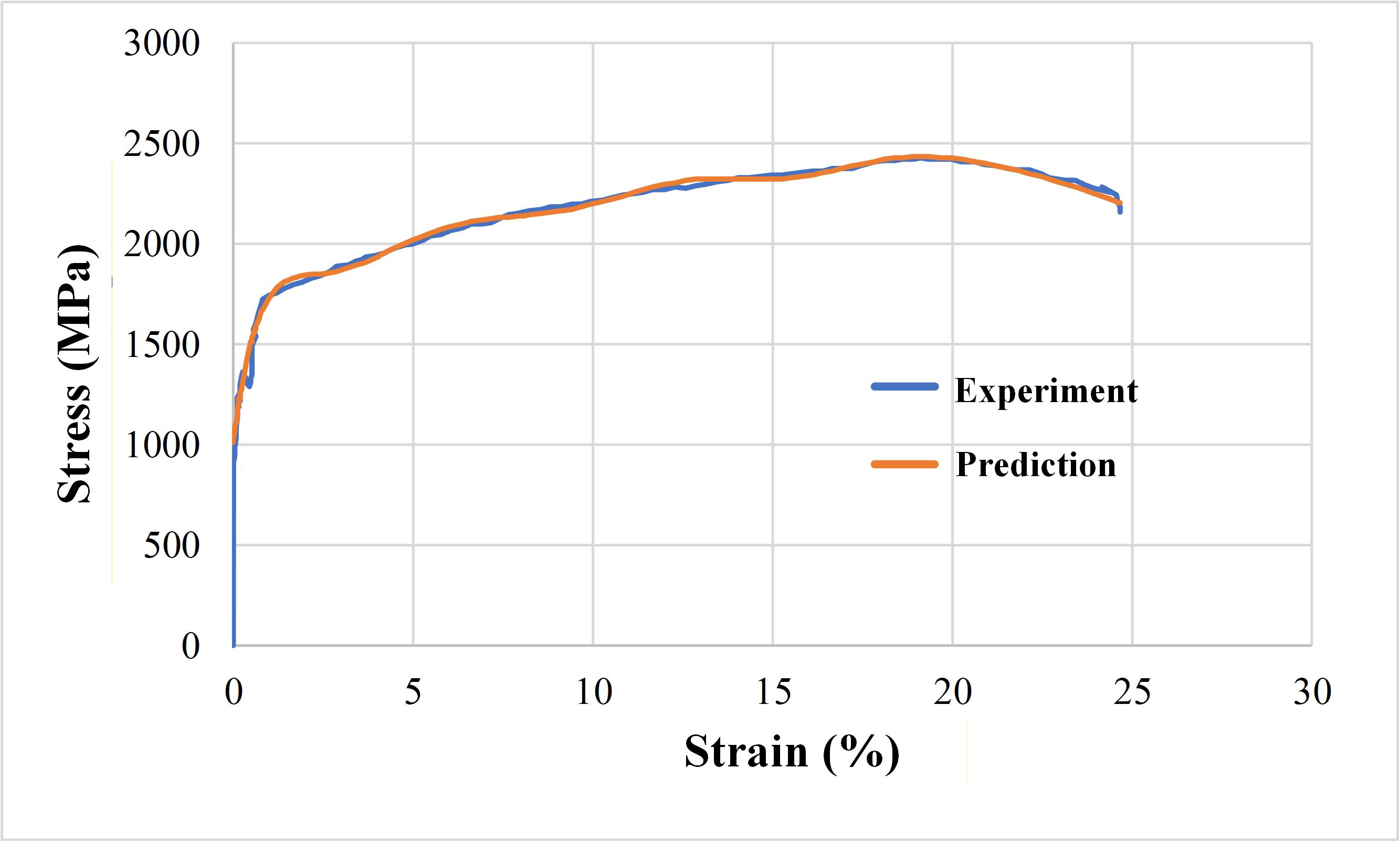

Supplement: S19 Fig — (JPG) [file pone.0349828.s019.jpg]

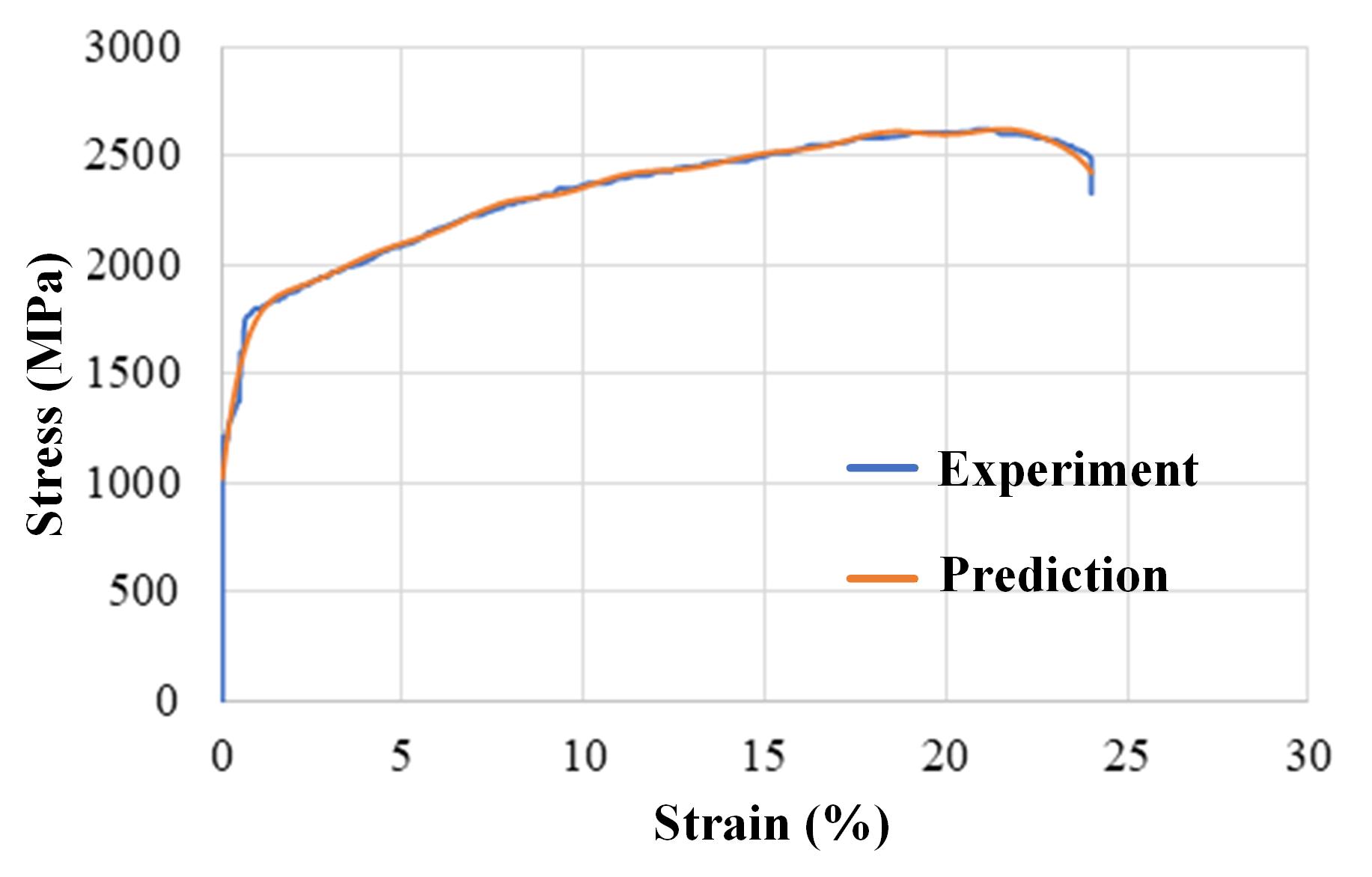

Supplement: S20 Fig — (JPG) [file pone.0349828.s020.jpg]

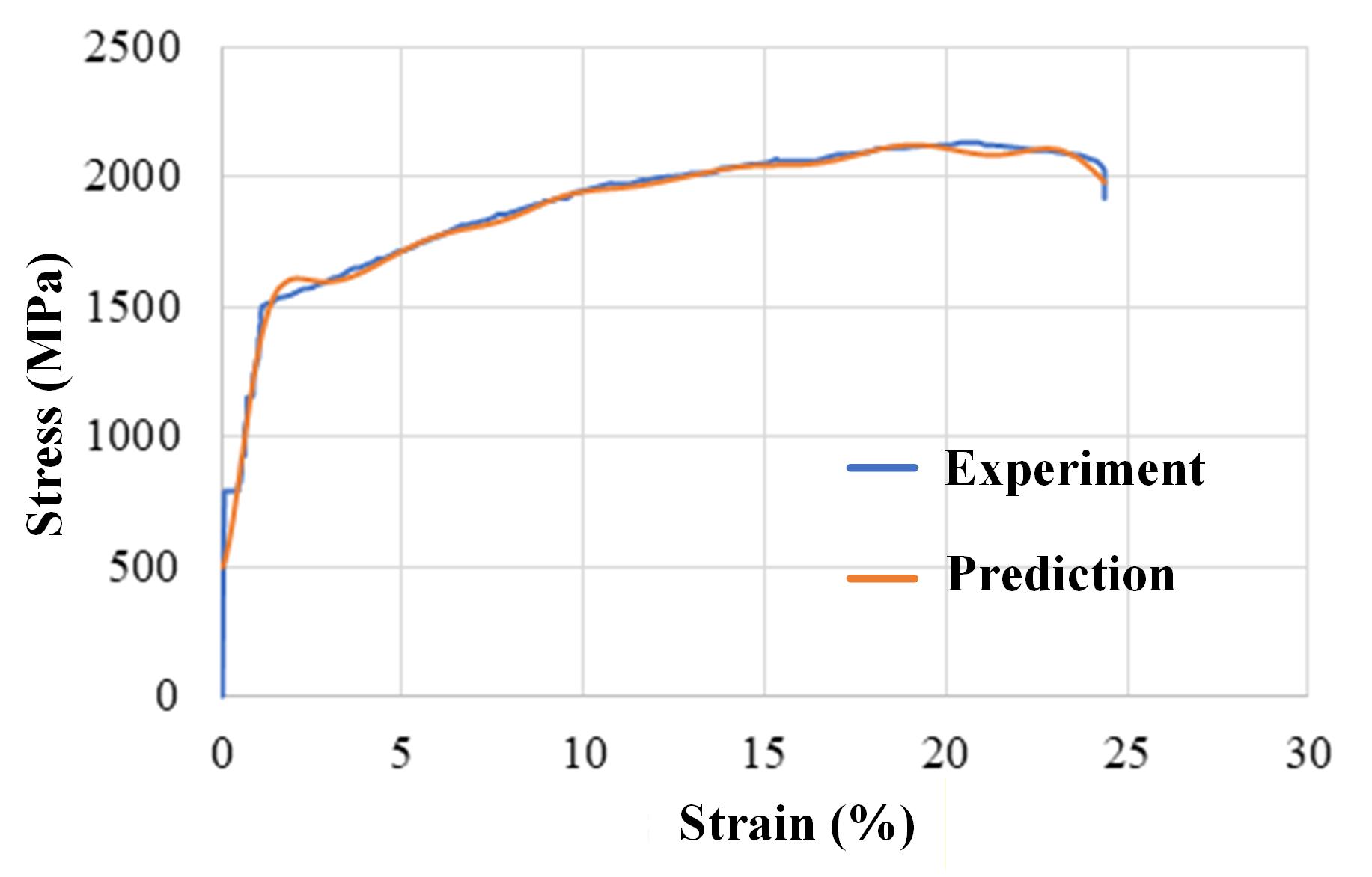

Supplement: S21 Fig — (JPG) [file pone.0349828.s021.jpg]

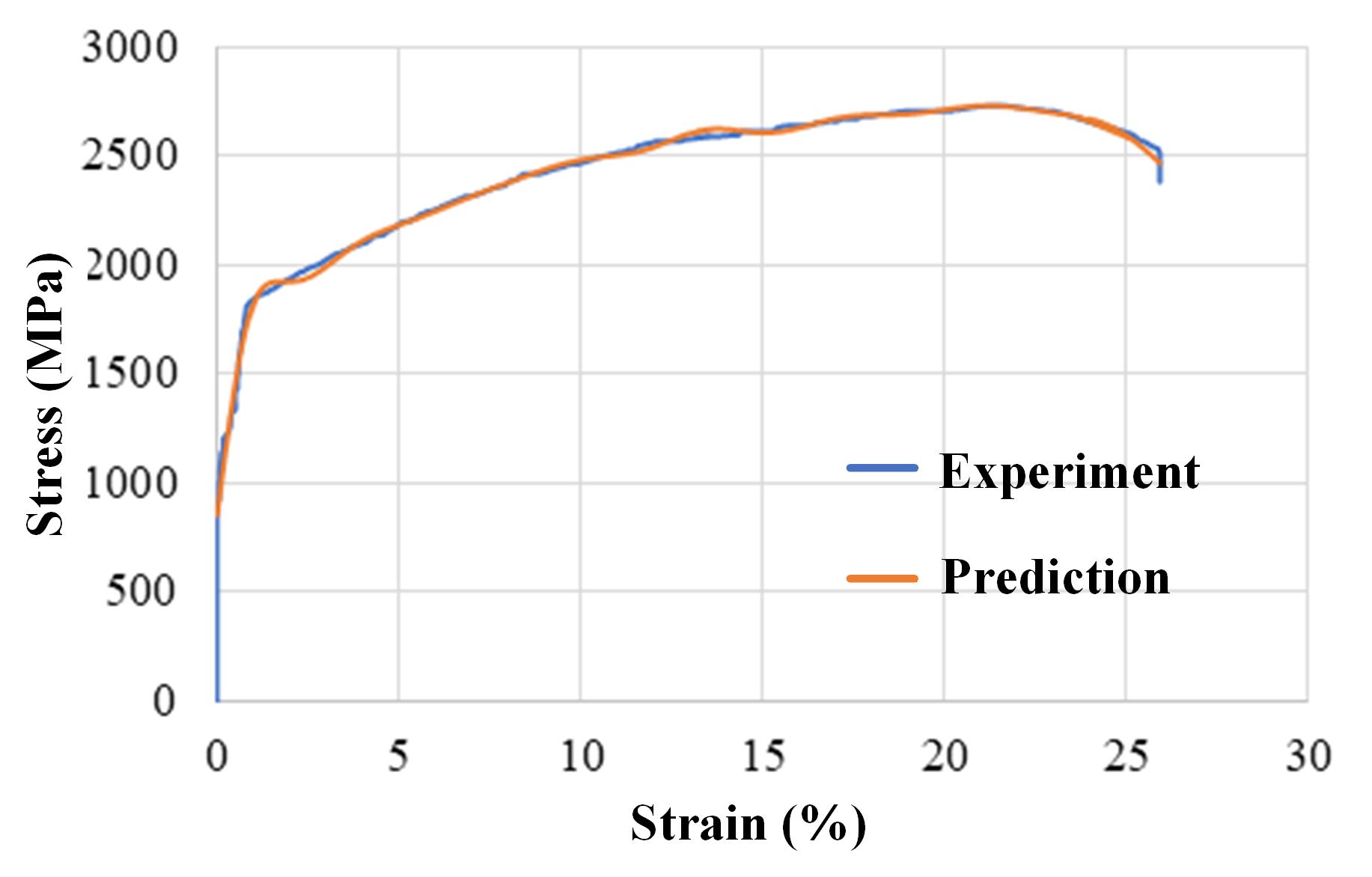

Supplement: S22 Fig — (JPG) [file pone.0349828.s022.jpg]

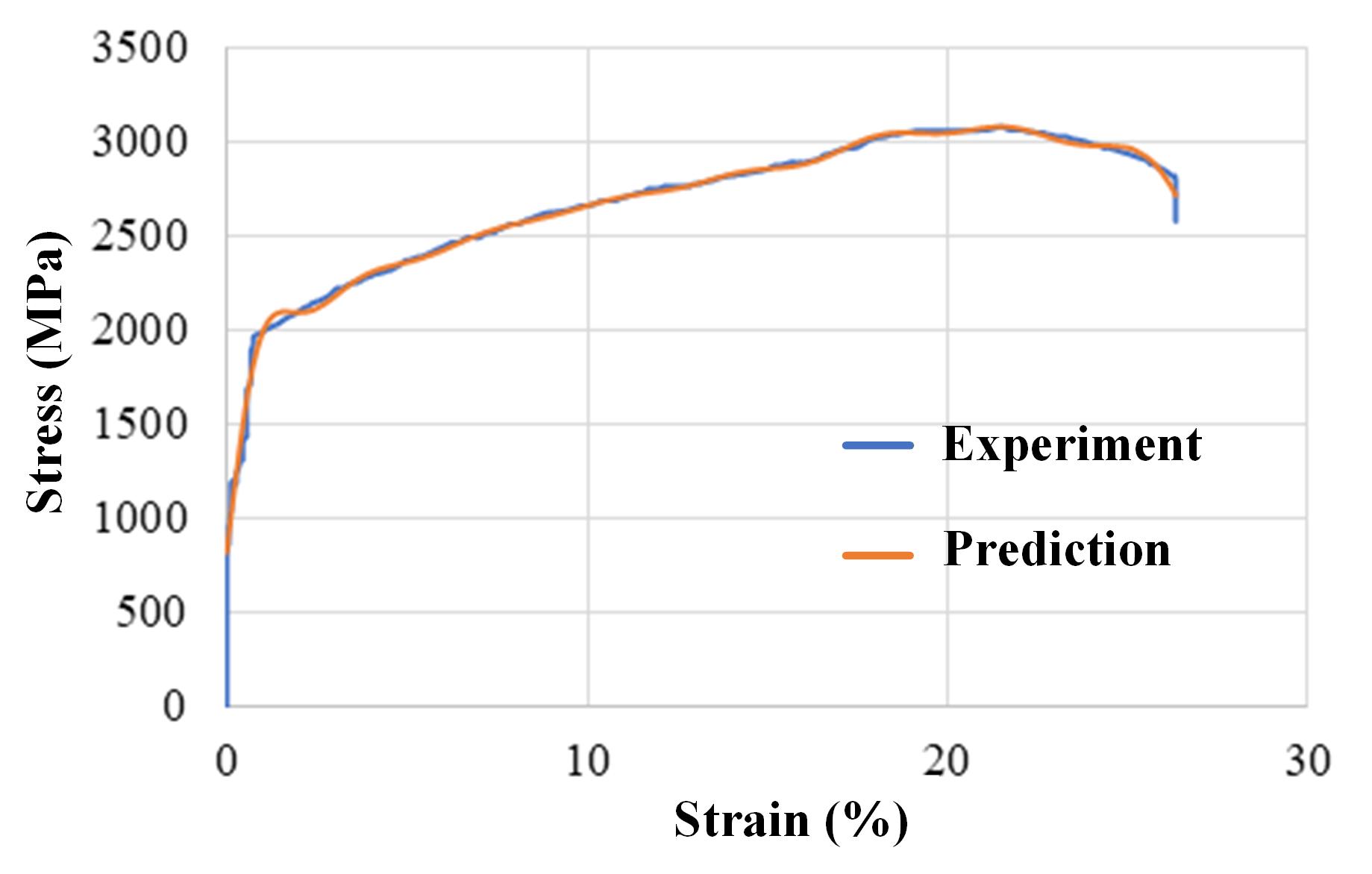

Supplement: S23 Fig — (JPG) [file pone.0349828.s023.jpg]

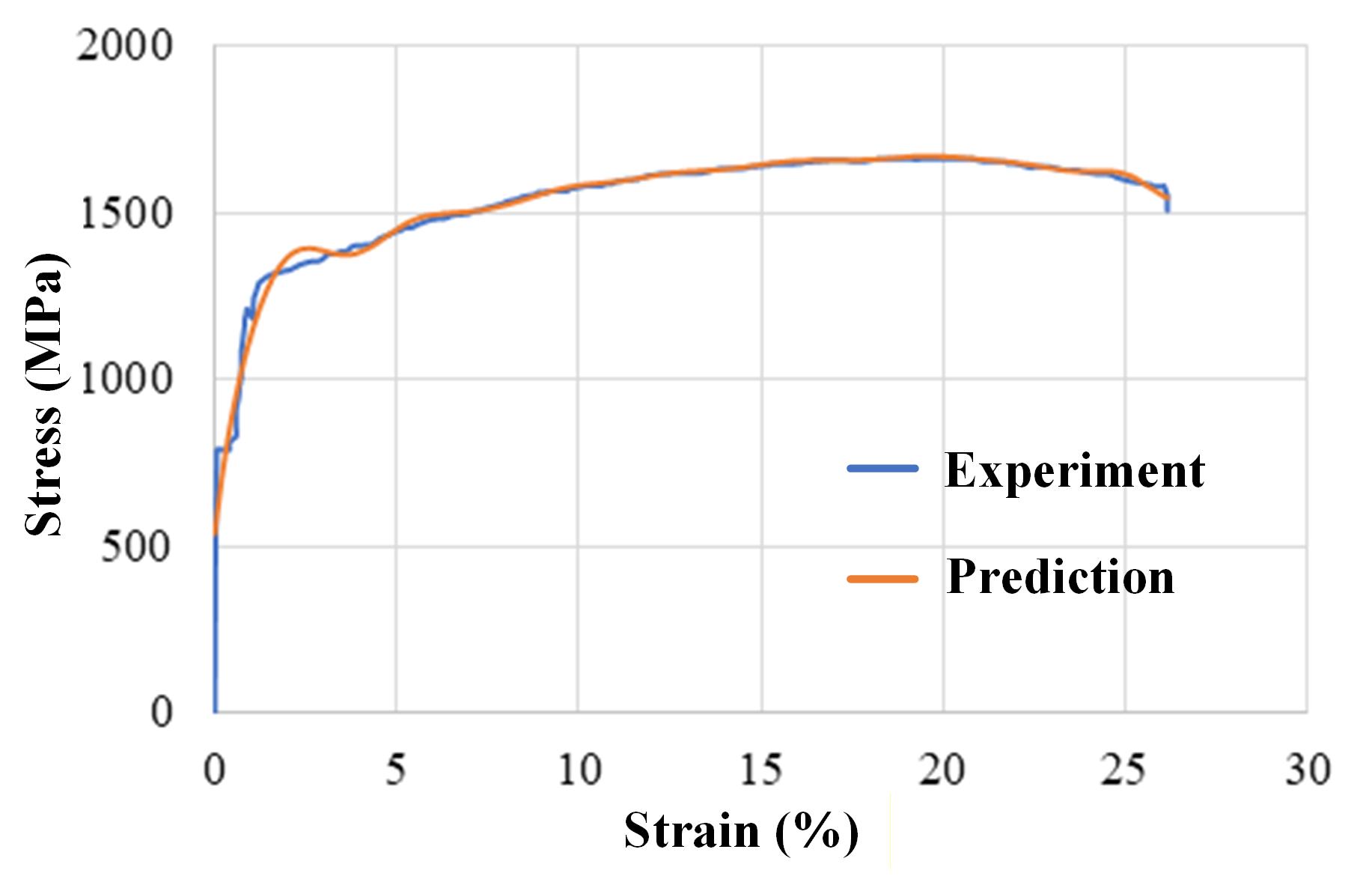

Supplement: S24 Fig — (JPG) [file pone.0349828.s024.jpg]

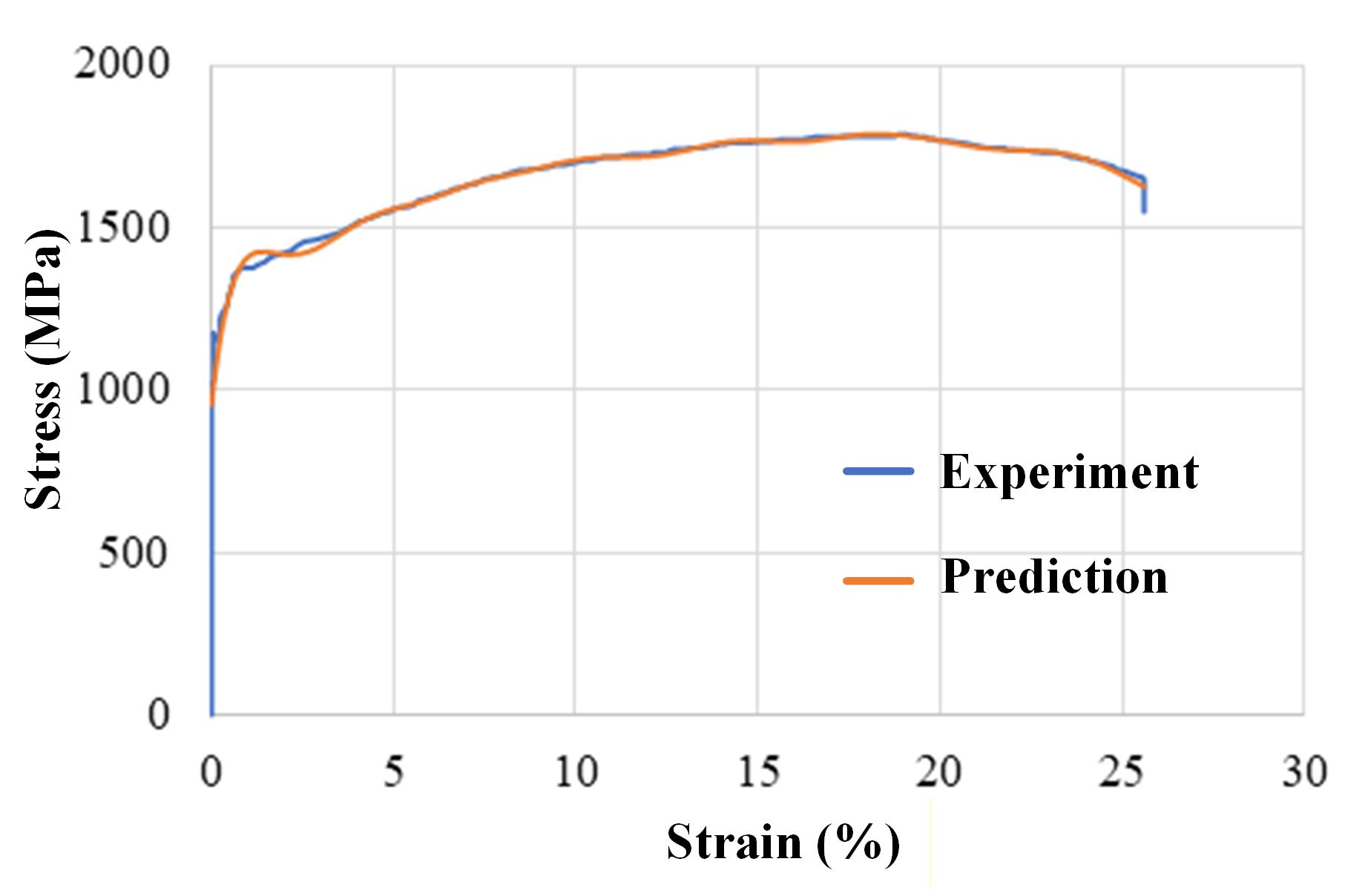

Supplement: S25 Fig — (JPG) [file pone.0349828.s025.jpg]

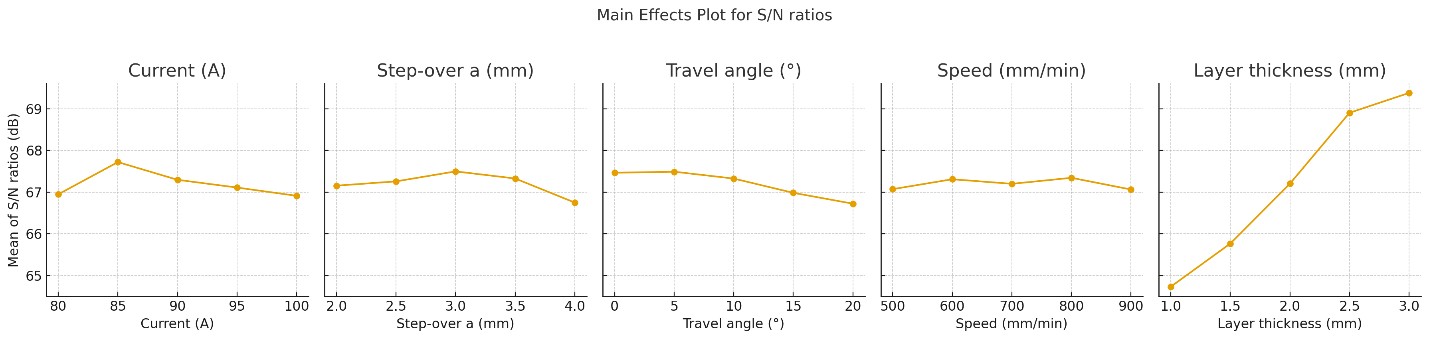

Supplement: S26 Fig — This is a main effects plot showing that layer thickness has the strongest influence on the S/N ratio of flexural strength in EF-WAAM specimens. (JPG) [file pone.0349828.s026.jpg]

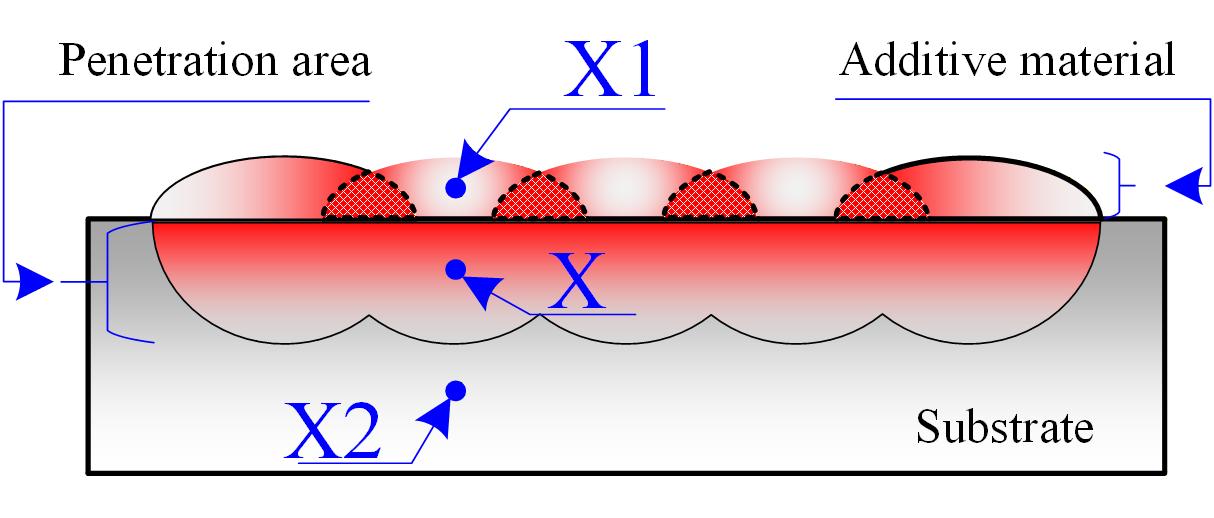

Supplement: S27 Fig — This is a schematic illustration showing the chemical composition measurement positions in the EF-WAAM sample, including the additive material, penetration area, and substrate. (JPG) [file pone.0349828.s027.jpg]
