# Supplementary material for: Edge-focused wire arc additive manufacturing: Method development with ANN-based stress–strain and mass-efficiency
Source: PLoS One. 2026 Jun 5;21(6):e0349828. doi: 10.1371/journal.pone.0349828 (PMC13240928; doi:10.1371/journal.pone.0349828)
Supplement: Attachment — Submitted filename: Reply_Reviewer 2.docx [file pone.0349828.s031.docx]

Dear Reviewer #2,

Thank you very much for your careful review of our revised manuscript and for your constructive comments. We sincerely appreciate your statement that the previous comments have been successfully addressed. We have carefully revised the manuscript according to your additional comments.

**Comment 1:**

Flowchart in Figure 11 need to be revised. At current form, no START and END included and need to use proper shapes to represent the activities.

**Response:**
Thank you very much for your valuable comment. Figure 11 has been revised accordingly. In the revised version, **START** and **END** symbols have been added. In addition, standard flowchart symbols were used to represent different types of activities more appropriately, including process steps, outputs, and decision-related actions.

The workflow was also reorganized to more clearly illustrate the experimental procedure, the comparison of bending strength among the three specimen groups, and the ANN-based prediction process. These modifications make the flowchart clearer, more complete, and easier to follow.

**Comment 2:**

References are outdated. Less than 50% of the references published within 5 years.

**Response:**
Thank you very much for pointing this out. We have carefully reviewed and updated the reference list. Several outdated references were replaced with recent SCIE papers that are closely related to the same research contents, including wire arc additive manufacturing, WAAM process parameters, residual stress and distortion, mechanical properties of WAAM steel, Taguchi optimization, ANN/machine learning prediction, and architectured materials.

In the revised manuscript, more than 80% of the references are now from the last five years. We also prioritized recent SCIE publications and relevant PLOS ONE papers where appropriate. This revision improves the timeliness and scientific support of the manuscript.

Once again, we sincerely thank you for your constructive comments, which helped us improve the quality and clarity of the manuscript.

Sincerely yours,

The Authors
